# Identification of a new C-terminal splice variant of Ca_V_1.3 L-type calcium channels with unique functional properties

**DOI:** 10.1186/1471-2210-11-S2-A44

**Published:** 2011-09-05

**Authors:** Gabriella Juhasz-Vedres, Anja Scharinger, Mathias Gebhart, Perrine Busquet, Chiara Poggiani, Simone B Sartori, Martina J Sinnegger-Brauns, Alexandra Koschak, Jörg Striessnig

**Affiliations:** 1 Department of Pharmacology and Toxicology, Institute of Pharmacy, and Center for Molecular Biosciences Innsbruck (CMBI), University of Innsbruck, 6020 Innsbruck, Austria

## Background

In L-type voltage-gated calcium channels (VGCCs) the long C-terminal tail contains several sites for modulation by protein-protein interaction. Ca_V_1.3 VGCCs (Ca_V_1.3_L_) activate at negative voltages and support sinoatrial node pacemaking and hearing, and shape neuronal excitability. In Ca_V_1.3_L_ an intermolecular automodulatory C-terminal interaction (CTM) has been described which affects channel gating. CTM is characterized by interaction of a distal C-terminal regulatory domain (DCRD) with a more proximal regulatory domain (PCRD). If this CTM is absent as in previously described short Ca_V_1.3_42A_, calcium-dependent inactivation (CDI) increases and the channel activation range shifts to more negative voltages (i.e. “short” gating properties). Here we show that alternative splicing in exon 43 creates a new short splice variant Ca_V_1.3_43S_ found in human and mouse brain. It lacks CTM, but still contains the PCRD motif, in contrast to previously described Ca_V_1.3_42A_. Semiquantitative PCR experiments showed that in mouse brain 39% of Ca_V_1.3 channels contain exon 43S contrary to heart (6% 43S).

## Methods and results

Biophysical analysis showed “short” gating properties for Ca_V_1.3_43S_ in both 15 mM and 2 mM external Ca^2+^ when co-expressed with β3 and α2δ-1 subunits in tsA-201 cells. In 2 mM Ca^2+^ the inactivation rate of Ca_V_1.3_43S_ was faster for Ca_V_1.3_L_, but slower for Ca_V_1.3_42A_ (% inactivation after 100 ms at V_max_: Ca_V_1.3_42A_: 64.5 ± 3.5%; Ca_V_1.3_43S_: 52 ± 4.5%, Ca_V_1.3_L_: 37.4 ± 3%, p < 0.001) by affecting the extent of CDI. Due to a presence of PCRD, DCRD-containing C-terminal fragments from Ca_V_1.3 or Ca_V_1.2 channels could restore Ca_V_1.3_L_ gating behaviour. Indeed, co-expression of GFP-Ca_V_1.2_C349_ fully restored long channel gating properties in Ca_V_1.3_43S_ (V_0.5_: Ca_V_1.3_43S_+GFP-Ca_V_1.2_C349_: 1.37 ± 1.3 mV; Ca_V_1.3_L_: −2.4 ± 0.6 mV). C-terminal splicing also changed I_Ca_ kinetics during stimuli mimicking trains of action potential waveforms, revealing a lower total I_Ca_ during AP bursts in short splice variants.

## Conclusions

Taken together, our data indicate that Ca_V_1.3 C-terminal splicing can serve as an important mechanism to fine-tune the dynamics of calcium entry in neurons in an activity dependent manner.

